# Development, preparation, and evaluation of a novel non-adjuvanted polyvalent dermatophytes vaccine

**DOI:** 10.1038/s41598-022-26567-3

**Published:** 2023-01-04

**Authors:** Heidy Abo-Elyazeed, R. Soliman, H. Hassan, F. R. El-Seedy, Hassan Aboul-Ella

**Affiliations:** 1 Department of Microbiology, Faculty of Veterinary Medicine, Cairo University, Giza, Egypt; 2 Animal Health Research Institute, Giza, Egypt; 3 Department of Microbiology, Faculty of Veterinary Medicine, Beni-Suef University, Beni-Suef, Egypt

**Keywords:** Immunology, Microbiology

## Abstract

Ringworm is a worldwide distributed contagious disease infecting both man and animals that constitute an economic, zoonotic, and health problem concern all over the world. During the last decade, attention has been directed to vaccination as an ideal approach to the control of such diseases. In the present study, non-adjuvanted polyvalent vaccines were prepared from locally isolated hot and virulent dermatophyte species, namely *Trichophyton verrucosum *(*T. verrucosum*)*, Trichophyton mentagrophytes *(*T. mentagrophytes*), and *Microsporum canis *(*M. canis*) were immunologically evaluated*.* The prepared vaccine evaluation was focused on the aspects of immunogenicity and protective efficacy using guinea pigs. Both in its living or inactivated forms, the vaccine-induced significant humoral and cell-mediated immune responses and achieve proper protection of guinea pigs against challenging infections with homologous and heterologous dermatophyte strains. On the other hand, investigations on dermatophyte exo-keratinases showed that it was better produced and more expressed in a mineral-based medium containing pure keratin (3 g/L) than in the same medium with human hair supplementation (2.6 g/L). The maximum dermatophyte productivity of exo-keratinases was found to be between 18 and 21 days post-incubation. Using sodium dodecyl sulfate–polyacrylamide gel electrophoresis (SDS-PAGE), two fractions with molecular weights of 40 kDa (fraction I) and 28 kDa (fraction II) have been identified in the culture filtrate of the three involved dermatophyte species. Both fractions demonstrated keratinolytic activity. The specific activity of the isolated keratinases (number of Keratinase units (KU)/mg protein) was stronger in fraction I, where it reached 18.75, 15.38, and 14 KU/mg protein as compared to 12.9, 8.74, and 12 KU/mg protein in fraction II of *T. verrucosum, T. mentagrophytes, and M. canis*, respectively. The dermatophyte exo-keratinases proved to be immunogenic as they stimulated high keratinase-specific antibody titers and induced strong delayed skin hypersensitivity reactions in vaccinated animals. Anti-keratinase-specific IgG was detected in sera of guinea pigs immunized with the inactivated or living polyvalent dermatophyte vaccines by a homemade enzyme-linked immunosorbent assay (ELISA) using dermatophyte exo-keratinases as coating antigen. The intradermal injection of dermatophyte exo-keratinases induced specific delayed skin reactions in guinea pigs immunized with the inactivated or the living polyvalent dermatophyte vaccines. The intradermal injection of dermatophyte exo-keratinases in the control non-sensitized guinea pigs was associated with itching, swelling, and bloody scar formation, however, no skin indurations were formed. The development of those post-exo-keratinases injection reactions in the control non-sensitized apparently healthy guinea pigs group, suggests an exo-keratinases possible role in the pathogenesis of dermatophytosis.

## Introduction

Dermatophytes as a closely related keratinolytic group of fungi with special mentioning of *T. mentagrophytes,* and *M. canis* representing the zoophilic groups; *T. benhamiae* complex and *M. canis* complex respectively as well as which are considered the most predominantly isolated dermatophyte species from animals infected with superficial mycosis worldwide. Also, *T. verrucosum* another member of the *T. benhamiae* complex with less worldwide distribution due to the increased vaccination-based control protocols but still predominantly existed in the Middle East. Dermatophytosis represent an economically important health problem in both productive and pet animals and on the other hand a serious zoonotic threat to human, particularly children and especially nowadays, due to the habitually increased animal-human companionships^1–14^.

Dermatophytosis is still considered a medical issue due to certain diagnostic complexities appropriate curative treatment selection difficulties, and a suitable case-oriented treatment protocol application period guarantee the overall threat of infection spread either human–human infectious or contagious based or animal-human zoonotic based spreading. Therefore, a proper control technique seems to be an ideal approach to avoiding active dermatophytes cases dealing with obstacles^15–18^. Several studies have attempted to develop vaccine-based dermatophyte control strategies based on active immunization against dermatophyte infection in animals using killed or living attenuated dermatophyte vaccines^15,19–24^. In Norway, a vaccine containing an attenuated strain of *T. verrucosum* is used against cattle ringworm since 1980. It stimulates humoral and cellular immune responses conferring protection against the disease. Vaccination campaigns in densely populated countries have contributed to a substantial decrease in the number of ringworm outbreaks^15^. Contradictory results, however, have been reported in different countries regarding the efficacy of the dermatophyte vaccines^25–27^.

The dermatophyte keratinases, on the other hand, seem to play an important role in the pathogenesis and immunity against dermatophytosis^28–34^. Also, attempts have been made to prepare dermatophyte subunit vaccines based on current knowledge about dermatophytes virulence factors like keratinases and their potential role in disease development, but with limited success so far^15^. An *M. canis* recombinant 31.5 kDa keratinase and a crude exo-antigen were evaluated as vaccines in an experimental infection model in guinea pigs. Vaccination induced remarkably high and significant antibody responses and high cell-mediated immune responses towards both antigens. After the challenge, however, scores reflecting the severity of dermatophyte lesions did not differ significantly between vaccinated and control groups at any time after the challenge^35^.

Despite the availability of effective vaccines against several microbial agents, vaccination against fungal agents, and especially dermatophytosis-causing agents requires improvement and further development in both animals and humans. Therefore, the aim of the current study was the preparation and evaluation of the protective and immunizing efficacy of the newly developed non-adjuvanted polyvalent dermatophyte vaccines, prepared from the most commonly occurring and isolated dermatophyte species. Moreover, to highlight the role of dermatophytes keratinases in the dermatophytic immune-pathogenic cycle.

## Material and methods

### Dermatophyte strains

*Trichophyton verrucosum* str. Tv-96-3, *T. mentagrophytes* str. Tm-96-1, *Microsporum canis* str. Mc-97-5, and *Trichophyton rubrum* (*T. rubrum*) str. Tr-98-1 strains were obtained from the Department of Microbiology, Faculty of Veterinary medicine, Cairo University. These strains were isolated and identified from animal clinical active cases submitted for further confirmed mycological laboratory investigation in the same department as well as they were selected for the vaccine preparation according to the criteria reported by Brandebusemeyer, 1990^36^. According to these criteria, we found that dermatophyte vaccinal strain should be; isolated from badly infected animals, grow rapidly in vitro, forming copious amounts of fungal mats, and be rich in fungal microconidia, which are known to carry the potent immunogenic determinants of the dermatophytes.

### Preparation, separation, and lyophilization of dermatophyte cultures

*T. mentagrophytes* and *M. canis* were inoculated separately into 0.5L Sabouraud’s dextrose broth (OXOID) and incubated at 25 °C for 4 weeks, while *T. verrucosum* was inoculated into 0.5L Sabouraud’s dextrose broth supplemented by thiamine (HIMEDIA) and inositol (HIMEDIA) and incubated at 37 °C for 6 weeks. The obtained matt-submerged fungal growth was then separated using sterile gauze. The harvested fungal mats were lyophilized and ground under aseptic conditions to form a fine powder. The number of colony-forming units (CFU)/mg of the lyophilized dermatophyte powder was determined on Sabouraud’s dextrose agar (SDA) plates^37^.

### Preparation of non-adjuvanted polyvalent dermatophyte vaccines

Two dermatophyte vaccine preparations from each species were prepared, a living and an inactivated one. In the living vaccine form, the lyophilized powder from the three selected dermatophyte species was mixed and distributed in 1 ml vials in a dose of 6 × 10^6^ total CFU*/*vial (2 × 10^6^ CFU from each dermatophyte species. The inactivated vaccine was made in the same way and the inactivation was performed according to Rybnikar, et al*.* 1996^38^, using Gamma irradiation (400 krad). This killing dose was pre-determined by investigating the effect of varying doses of radio cobalt (100–400 krad) on dermatophyte viability through exposure-post-exposure culturing to confirm the killing efficiency and the minimal dose able to achieve that for the involved dermatophytes strains.

### Immunization of guinea pigs

Three groups of adult female guinea pigs were used in this experiment, each group consisted of three animals. The first group was inoculated with the inactivated vaccine, the second with the living vaccine, and the third one was left as unvaccinated control. Moreover, another ten-member based group apparently healthy unvaccinated guinea pigs group was kept with those immunized with the living vaccine (second group) as a contact control. In the first two groups, each animal was injected intramuscularly (I/M) twice, at 2 weeks intervals, with 0.2 ml suspension of the polyvalent vaccine containing 6 × 10^6^ CFU/ml. Two weeks after the second dose, the immunizing and the protective efficacies of the tested vaccine preparations were determined by measurement of the developed immune responses as well as by a challenge test.

Zero-day blood samples were collected from all animals involved in the study before vaccination to exclude ant asymptomatic cases and weekly after the priming vaccination dose, and of course after the challenge infection.

### Immune response evaluation testing

The specific antibody production representing humoral immunity was detected using a homemade ELISA^39,40^, and the cell-mediated immune response was determined using a Trichophytin skin test^41^.

#### Homemade dermatophyte ELISA development

An aqueous whole dermatophyte extract antigens prepared from the three dermatophyte species mentioned above as homologous antigens as well as from a *Trichophyton rubrum* (*T. rubrum*) strain as heterologous antigen, were used as plate coating antigens in the humoral immunity evaluating mentioned homemade ELISA^39^.

#### Post-vaccination challenging infection

All animals were subjected to challenge infection with 0.2 ml of 21 days old culture suspension of the four dermatophytes species on an area of the skin exactly on the following site; caudal thorax, where the hairs were clipped, and the skin surface was gently scratched with sterile sandpaper.

### Keratinase production investigation

*T. verrucosum, T. mentagrophytes,* and *M. canis* were investigated by inoculating a unified fungal suspension (5 × 10^6^) of each strain separately into a mineral medium enriched with human hairs (2.6 g/L) or keratin (3 g/L) and incubated for 30 days. The keratinolytic activity of the dermatophyte exo-keratinases activity was determined every 3 days according to Siesenop, U. 1998^42^. The characterization of the exo-keratinase was done using SDS-PAGE^36,43^.

### Studies of the immune response to dermatophyte exo-keratinase fractions

The humoral and cell-mediated immune responses developed against dermatophyte exo-keratinases in guinea pigs vaccinated with the living and inactivated dermatophyte vaccines were determined by homemade ELISA and Trichophytin skin tests using exo-keratinase fractions of the 4 dermatophyte species as antigens.

### Ethical statement

The current conducted study is reported in accordance with (Animal Research: Reporting of In-Vivo Experiments-ARRIVE) guidelines. All experimental protocols were approved by the Institutional Animal Care and Use Committee-IACUC of the faculty of veterinary medicine, at Cairo University. The guidelines of the (Institutional Animal Care and Use Committee-IACUC of the faculty of veterinary medicine, Cairo University) were completely followed during any procedures involving animal use through the current conducted study. No anesthesia or euthanasia protocols were used with the animal involved during this study as all animal-dependent methodological procedures were considered as no to low pain-causing procedures that ethically can be done on conscious alive animals.

## Results

### Protective efficacy of the non-adjuvanted polyvalent dermatophyte vaccines

**Figure 1:**
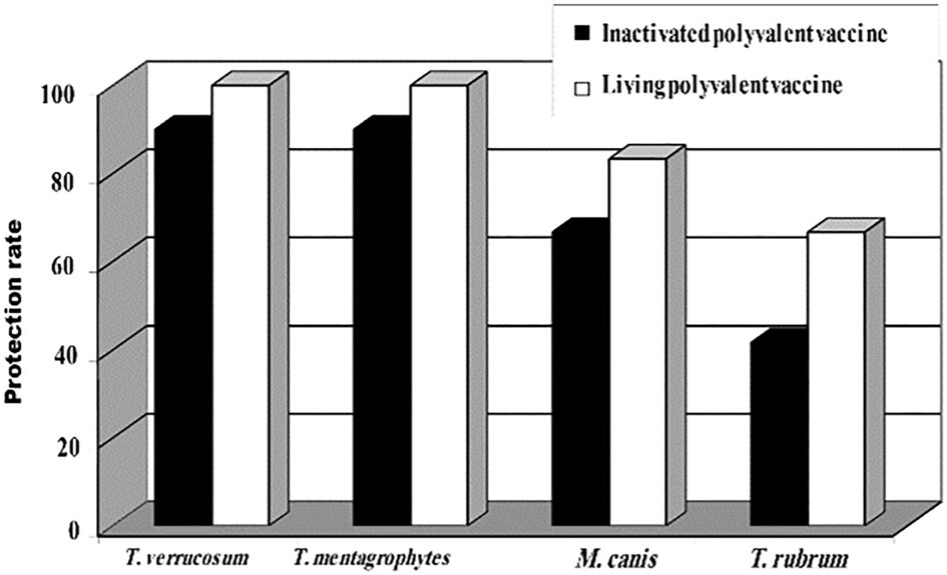
Protective efficacy of the inactivated and the living non-adjuvanted polyvalent dermatophyte vaccines against challenge with virulent dermatophyte species.

The non-adjuvanted inactivated polyvalent vaccine induced a protection rate of 90.0, 90.9, 66.97, and 41.67% against challenges with virulent strains of *T. verrucosum. T. mentagrophytes, M. canis and T. rubrum*, respectively. The protective efficacy of the non-adjuvanted living polyvalent vaccine was significantly higher than that of the inactivated one and reached 100, 100, 83.33, and 66.67 against challenge with *T. verrucosum. T. mentagrophytes, M. canis and T. rubrum*, respectively. Challenge infection of the non-immunized control animals with the same strains induced infection rates of 83.33%, 91.67, 100, and 100%, respectively, (Fig. 1).

Among guinea pigs immunized with the living polyvalent vaccine, only two animals developed ringworm lesions at the site of immunization. *M. canis* was isolated from both cases. On the other hand, contact non-immunized animals that were kept in the same cages remained apparently healthy during the observation period, which extended to 8 weeks post-challenge.

### Immune responses to the non-adjuvanted polyvalent dermatophyte vaccine

#### Humoral immune response

**Figure 2:**
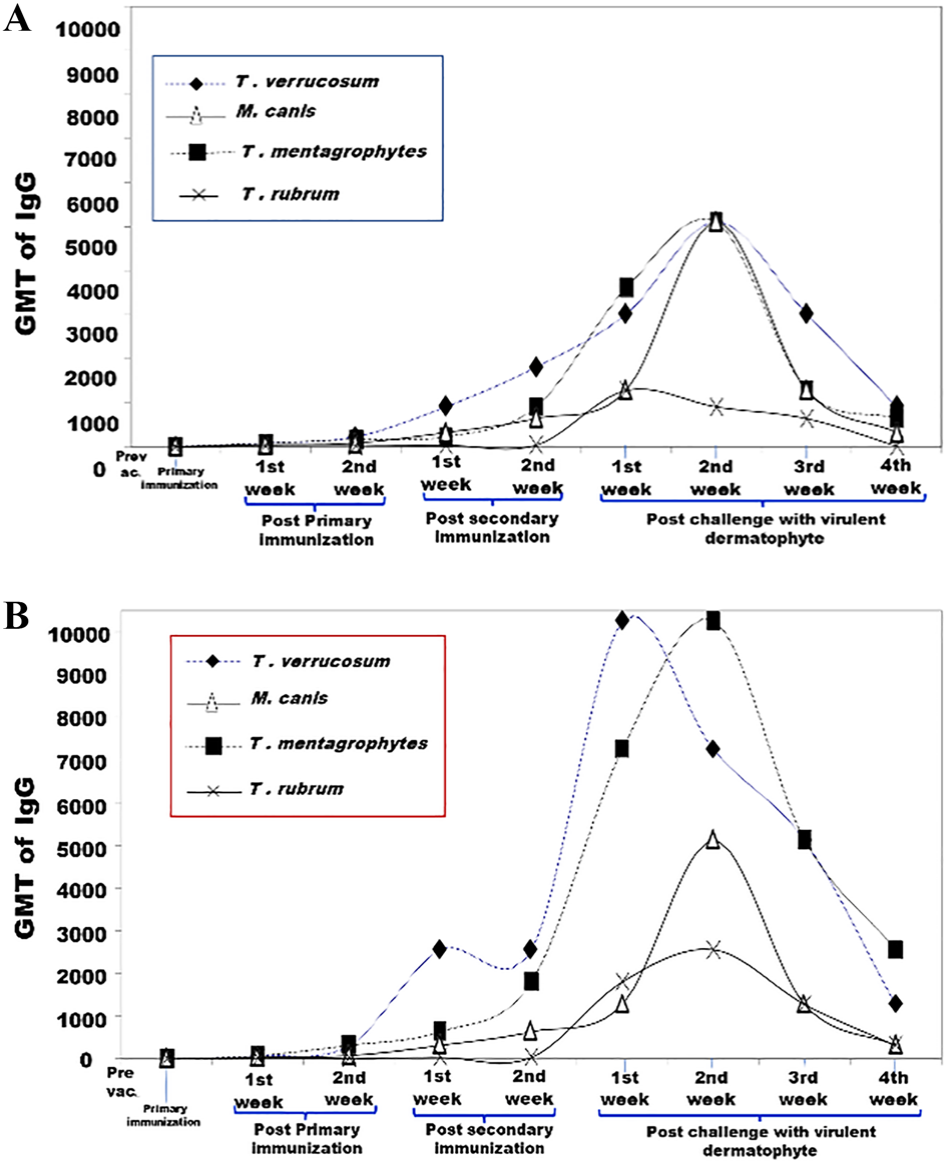
(**A**) ELISA GMT of anti-*T. verrucosum*, *T. mentagrophytes*, *M. canis*, and *T. rubrum* -specific IgG in sera of Guinea pigs immunized with the non- adjuvanted inactivated polyvalent dermatophyte vaccines; (**B**) ELISA GMT of anti-*T. verrucosum*, *T. mentagrophytes*, *M. canis*, and *T. rubrum* IgG in sera of Guinea pigs immunized with the non- adjuvanted living polyvalent dermatophyte vaccines.

Guinea pigs immunized with the non-adjuvanted inactivated or living polyvalent dermatophyte vaccines developed anti-dermatophyte specific IgG that was measured by ELISA. In guinea pigs immunized with the inactivated vaccine, the Geometric mean titers (GMT) of the IgG-specific antibody titers against *T. verrucosum, T. mentagrophytes, M. canis,* and *T. rubrum* were equal to 1810, 905. 640 and 40 ELISA units/ml, respectively, when measured 2 weeks post-second dose, a significant rise in the antibody titers were measured 2 weeks post-challenge with the virulent dermatophyte strains. The antibody titers reached 5120 units/ml against *T. verrucosum, T. mentagrophytes, M. canis,* and 905 units/ml against *T. rubrum* (Fig. 2A).

Significantly higher antibody titers were measured in guinea pigs immunized with the non-adjuvanted living polyvalent vaccine (Fig. 2B). The specific GMT of IgG measured 2 weeks after the second vaccinal dose reached 2560, 1810, 5120 and 40 ELISA units/ml against *T. verrucosum, T. mentagrophytes, M. canis* and *T. rubrum*, respectively. Further rise in the antibody titers was recorded 2 weeks post-challenge the GMT reached 7241 units/ml against *T. verrucosum* and 10,240 units/ml against *T. mentagrophytes*.

#### Cell-mediated immune response

**Figure 3:**
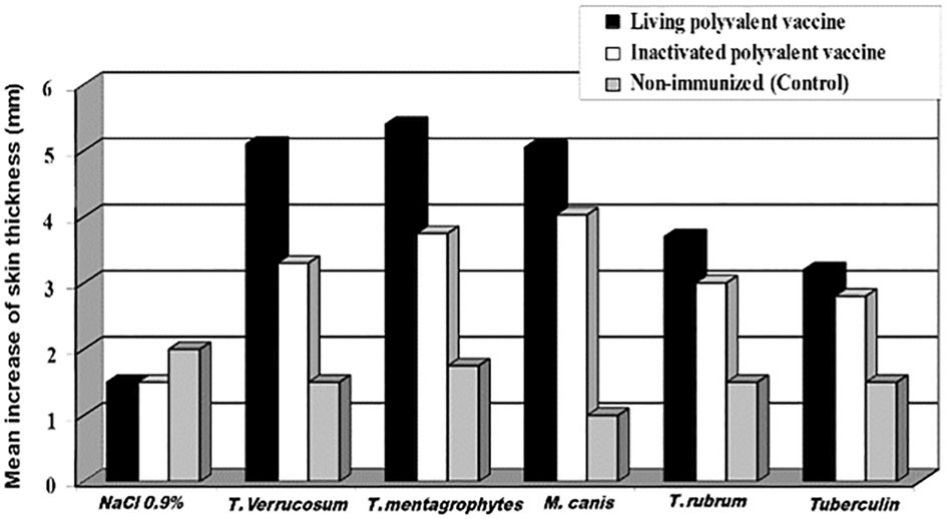
Illustrative chart of a single dermatophyte species based-Trichophytin skin test in Guinea pigs vaccinated with the living, inactivated non-adjuvanted polyvalent dermatophyte vaccines, and the control unimmunized group. Tuberculin test was done using *Mycobacterium bovis* purified protein derivatives (PPD) as non-specific antigen and NaCl 0.9% was used as negative control.

**Figure 4:**
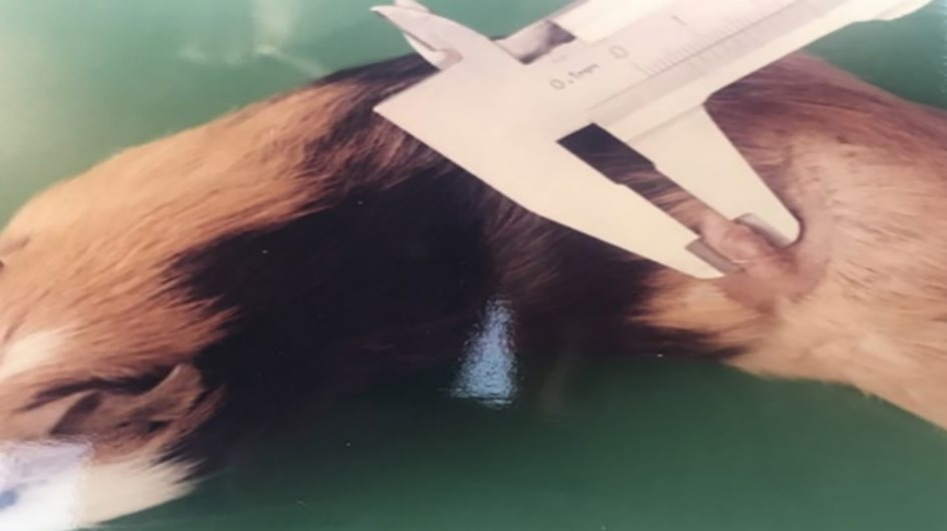
Trichophytin skin test in Guinea pig sensitized with non-adjuvanted polyvalent dermatophyte vaccine, typical delayed hypersensitivity skin reaction is illustrated.

Using the Trichophytin skin test, strong delayed hypersensitivity reactions were recorded against the homologous and heterologous dermatophyte Trichophytin in the immunized guinea pigs. The skin reactivity was more pronounced in those immunized with the living vaccine (Figs. 3 and 4).

### Isolation and characterization of dermatophyte exo-keratinases

**Figure 5:**
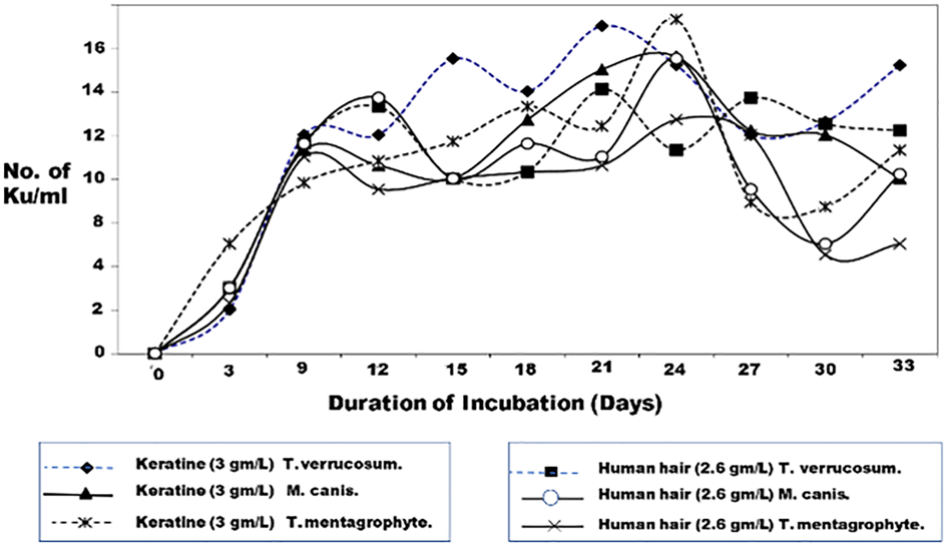
Correlation curve illustrates the effect of keratin source and incubation time on in vitro production of exo-keratinases (keratin unit/ml) by *M. canis*, *T. verrucosum*, and *T. mentagrophytes.*

**Figure 6:**
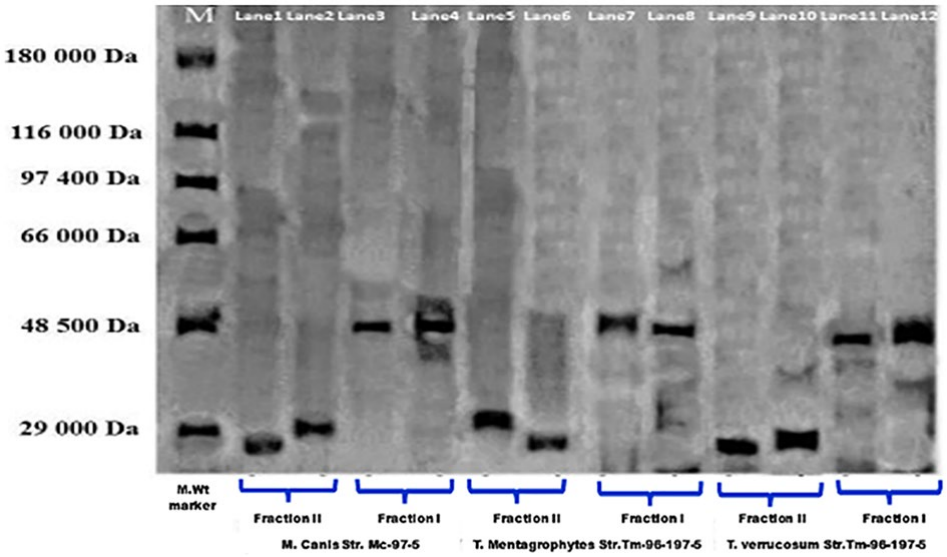
SDS-PAGE of culture supernatant of dermatophyte species grown on mineral medium containing 3 g/L keratin for identification of dermatophyte exo-keratinase based on the molecular weight. Lanes 1, 2, 5, and 6 showed 28 kDa bands (Fraction II) while 9 and 10 showed 42.5 kDa for the same fraction, and Lanes 3, 4, 7, and 8 showed 41 kDa bands (Fraction I), while 11 and 12 showed 27 kDa bands for the same fraction. (M) Indicating for the molecular weight protein ladder/marker (6500–180,000, Sigma-Aldrich). The molecular weight was estimated using a GELPRO3 analyzer.

Dermatophyte exo-keratinases were better produced in a mineral medium containing pure keratin (3 g/L) than in the same medium with human hair (2.6 g/L). The maximum production of exo-keratinases was found to be between 18 and 21 days post incubation (Fig. 5). Similar, if not identical fractionation patterns have been demonstrated with culture filtrates from the three dermatophyte species. The exo-keratinase fractions separated by the gel chromatography and monitored by SDS-PAGE revealed two bands in the culture filtrate of each dermatophyte. The first band corresponding to a molecular weight of about 40 kDa and the second fraction had a molecular weight of 28 kDa (Fig. 6), (Supplementary Figure S1).

### Biological activities of exo-keratinases

The specific keratinolytic activity of the purified dermatophyte exo-keratinase (the number of KU/mg protein) was determined. It was stronger in fraction I, where it reached 18.75, 15.38, and 14 KU/mg protein, as compared to 12.9, 8.74 and 12 KU/mg protein in fraction II of *T. verrucosum, T. mentagrophytes and M. canis*, respectively.

### Immunological activities of dermatophyte exo-keratinases

#### Humoral immune response

**Figure 7:**
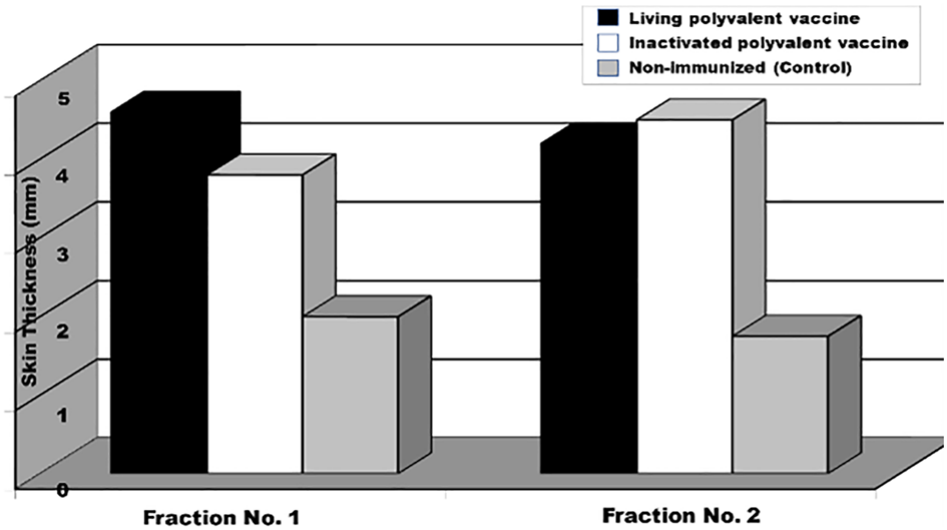
Delayed Skin reaction to dermatophyte exo-keratinase antigens (Fraction I and II) in guinea pigs immunized with the living or the inactivated non-adjuvanted polyvalent dermatophyte vaccines.

Using the heat-inactivated exo-keratinases as coating antigens in the ELISA test, anti-keratinase specific IgG was measured in sera of guinea pigs immunized with the non-adjuvanted inactivated or living polyvalent dermatophyte vaccines (Fig. 7). The anti-keratinase IgG antibodies increased slowly following vaccination with the inactivated dermatophyte vaccine and sharply two weeks post-challenge reaching to a maximum level of 1810 ELISA units/ml. The antibody titers were significantly higher in the sera of animals immunized with the living vaccine, where they reached a level of 2560 ELISA units/ ml, 2 weeks post challenge.

#### Cell-mediated immune response

The intradermal injection of dermatophyte exo-keratinases (pooled fraction I or pooled fraction II) induced specific delayed skin reaction in guinea pigs immunized with the non-adjuvanted inactivated or the living polyvalent dermatophyte vaccines (Fig. 7). The reaction was associated with the development of strong cellular reaction of delayed nature at sites of injection. This reaction involved itching, induration, and bloody scar formation. It had been observed also that the injection of the dermatophyte keratinases in the control non-sensitized guinea pigs induced inflammatory reaction associated with erythema, itching, and bloody crust formation, but without skin induration.

## Discussion

In the history of veterinary clinical dermatology, it has been observed that clinical dermatophyte infection is most often seen in young animals and following recovery or clearance of the original dermatophyte infection, re-infection is rare whether by the original dermatophyte species or by a different one^44^. These observation stands behind the repeated trials to develop active immunization against dermatophytosis in animals.

The aim of the present work was to develop a broad-spectrum polyvalent dermatophyte vaccine against animal ringworm, therefore, the most frequently isolated dermatophyte species from cattle and pet animals according to the frequency of their isolation in previous literature, namely, *T. verrucosum, T. mentagrophytes,* and *M. canis* were selected as a candidate for this vaccine.

Two types of non-adjuvanted polyvalent dermatophyte vaccines were prepared and their immunizing and protective efficacies were evaluated in a guinea pig model. The first vaccine was inactivated by gamma radiation^38,45^ where a dose of 400 k rad induced complete inactivation of the three dermatophyte species. The non-adjuvanted inactivated polyvalent dermatophyte vaccine, prepared from the three above-mentioned dermatophyte species, protected guinea pigs against challenge infection with virulent homologous strains (66.67–100%). The obtained results agreed with what has been reported by several previously conducted studies in the same field^21–24,46–49^.

It is worth to mention that a protection rate of 41.67% has been recorded when vaccinated guinea pigs were challenged with a heterologous dermatophyte species, namely, the *T. rubrum* strain. This dermatophyte species induced a 100% infection rate among the control non-immunized animals. The recorded cross-protection might be attributed to the cross-antigenic relationship between different dermatophyte species^44,50,51^. The living polyvalent vaccine was significantly more protective than the inactivated one, a result, which is comparable with those reported by other researchers^15,20^.

In contrast to the inactivated dermatophyte vaccine, which did not induce any adverse side effects on the immunized guinea pigs, the use of the living vaccine was, however, associated with certain disadvantages, as two of the immunized animals developed clinical manifestations of ringworm. The recovered dermatophyte species in these cases was *M. canis*. The failure of *M .canis* as a protective antigen has also been reported previously by DeBoer et al*.* 2002^25^. The adverse side effects of the living vaccine and the possibility of inducing infection certainly detract from its protective value. However, the lesions associated with the use of the living vaccine, if occurred, are mild and the infected animals undergo rapid recovery. The process of lypholization of the dermatophyte fungal mass during the vaccine preparation together with the intramuscular route of injection of the living dermatophyte vaccine significantly reduces the viability and virulence of the dermatophyte species^52^. The use of the inactivated dermatophyte vaccines in the control of dermatophytosis is recommended by Westhoff et al., 2010^21,22^ because of its proven safety.

In addition to its protective efficacy, the tested dermatophyte vaccine induced significant humoral and cellular immune responses. Several authors have documented the production of humoral and cellular immune responses in animals following vaccination or infection by dermatophytes^15,20,39,40,51,53,54^. The role of the immune responses in the clearance of an active infection or resistance to upcoming dermatophyte infections has been reported by several studies^20,47,51,55–60^.

The challenge of the immunized animals with different virulent dermatophytes was associated with a significant rise in antibody titers^39^. This increase was also significant when the challenge was made by a heterologous dermatophyte, *T. rubrum*. This is of particular importance, as it documents the strong cross-antigenic relationship between dermatophytes and the possible broad-spectrum protective value of the prepared vaccine against a long list of dermatophytes species rather than those actually used in vaccine preparation^50^.

According to Selvam et al*.* 2012^61^ there are several important biotechnological applications of microbial keratinase, and dermatophyte keratinases are considered as a possible promising candidate for prophylactic and therapeutic application against dermatophytosis. The dermatophyte keratinases have been identified and studied by several investigators^31,32,34,35,37,62–67^. In the current work, the dermatophyte exo-keratinases produced by *T. verrucosum, T. mentagrophytes,* and *M. canis* proved to be highly immunogenic as indicated by the induction of high antibody titers and strong delayed skin hypersensitivity reaction in vaccinated animals. Comparable results have been recorded in the following studies^27,35,68–70^.

The development of inflammatory reaction, itching, and bloody scar formation in the apparently healthy non-immunized animals injected with dermatophyte exo-keratinases explains its possible role in the inflammatory reaction and the itching associated with dermatophytosis. This reaction differs, however, from that recorded in the immunized animals, which manifested skin induration typical to the specific delayed hypersensitivity reaction. In the Trichophytin skin test, all Trichophytin preparations from homologous or heterologous dermatophyte species induced delayed hypersensitivity reaction in vaccinated guinea pigs. This finding indicated the possibility of presence of dermatophyte group- specific antigen(s) on which those cross-reactivity reaction were occurred.

To conclude, developing a polyvalent dermatophyte vaccine showed a promising protective prophylactic choice that is able to stand against dermatophytosis, with no or minimal post-vaccination reaction in the case of inactivated and living vaccines, respectively (Supplementary Information).

## Supplementary Information

Supplementary Figure S1.

## Data Availability

The datasets used and/or analyzed related to the animal cases tested during the current study are available from the corresponding author on reasonable request.
