# Supplementary material for: Development, preparation, and evaluation of a novel non-adjuvanted polyvalent dermatophytes vaccine
Source: Sci Rep. 2023 Jan 4;13:157. doi: 10.1038/s41598-022-26567-3 (PMC9813246; doi:10.1038/s41598-022-26567-3)
Supplement: Supplementary file 1 — Supplementary Figure S1. [file 41598_2022_26567_MOESM1_ESM.docx]

**
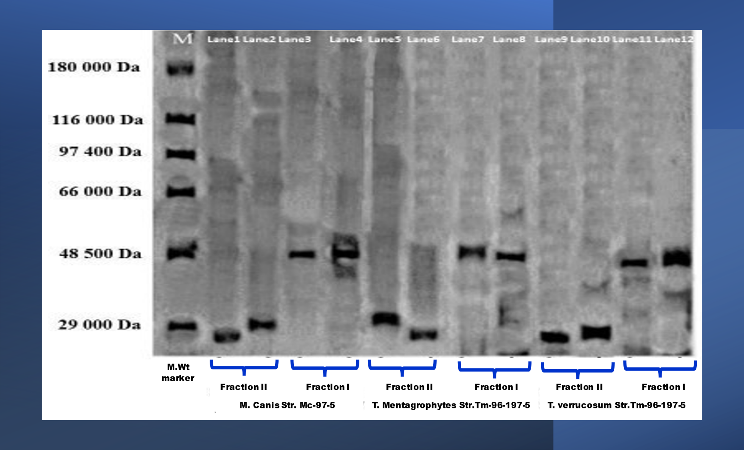
**

**Figure S1: SDS-PAGE of culture supernatant of dermatophyte species grown on mineral medium containing 3g/L keratin for identification of dermatophyte exo-keratinase based on the molecular weight. Lanes 1, 2, 5, and 6 showed 28 kDa bands (Fraction II) while 9 and 10 showed 42.5 kDa for the same fraction, and Lanes 3, 4, 7, and 8 showed 41 kDa bands (Fraction I), while 11 and 12 showed 27 kDa bands for the same fraction. (M) Indicating for the molecular weight protein ladder/marker (6500-180.000, SIGMA-ALDRICH). The molecular weight was estimated using a GELPRO3 analyzer.**
